# The relative contribution of acoustic signals versus movement cues in group coordination and collective decision-making

**DOI:** 10.1098/rstb.2023.0184

**Published:** 2024-06-23

**Authors:** Chun-Chieh Liao, Robert D. Magrath, Marta B. Manser, Damien R. Farine

**Affiliations:** ^1^ Division of Ecology and Evolution, Research School of Biology, Australian National University, Canberra, ACT , 2600, Australia; ^2^ Department of Evolutionary Biology and Environmental Studies, University of Zurich, Zürich , 8057, Switzerland; ^3^ Department of Collective Behavior, Max Planck Institute of Animal Behavior, Radolfzell , 78315, Germany

**Keywords:** collective behaviour, group cohesion, movement, quorum, vocal communication

## Abstract

To benefit from group living, individuals need to maintain cohesion and coordinate their activities. Effective communication thus becomes critical, facilitating rapid coordination of behaviours and reducing consensus costs when group members have differing needs and information. In many bird and mammal species, collective decisions rely on acoustic signals in some contexts but on movement cues in others. Yet, to date, there is no clear conceptual framework that predicts when decisions should evolve to be based on acoustic signals versus movement cues. Here, we first review how acoustic signals and movement cues are used for coordinating activities. We then outline how information masking, discrimination ability (Weber’s Law) and encoding limitations, as well as trade-offs between these, can identify which types of collective behaviours likely rely on acoustic signals or movement cues. Specifically, our framework proposes that behaviours involving the timing of events or expression of specific actions should rely more on acoustic signals, whereas decisions involving complex choices with multiple options (e.g. direction and destination) should generally use movement cues because sounds are more vulnerable to information masking and Weber’s Law effects. We then discuss potential future avenues of enquiry, including multimodal communication and collective decision-making by mixed-species animal groups.

This article is part of the theme issue ‘The power of sound: unravelling how acoustic communication shapes group dynamic’.

## 1. Introduction

To maximize the benefits of group living, social animals must maintain cohesion and coordinate their activities. This necessitates mechanisms that allow groups to express the same level of behavioural flexibility as individuals yet in a coordinated manner. Group behaviours can include small-scale shifts, such as switching from resting to foraging [1], through to large-scale movements, such as collective migration [2]. Understanding how social animals maintain cohesion, coordinate their actions and influence the group for their own needs—especially while navigating dynamic environments and changing circumstances—is fundamental to unravelling the evolution of animal societies [3].

What actions should we take? Where and when should we go? As humans, we constantly engage in consensus decision-making. And, we are not alone in facing the challenges of making consensus decisions; many other group-living animals also rely on making consensus decisions and coordinating their actions in order to function as a group. One of the major challenges associated with reaching consensus is that groups generally comprise individuals with differing needs and capabilities [4]. Having some means of communication, therefore, often plays a critical role in reducing consensus costs among group members by allowing them to rapidly coordinate their behaviours [5]. For instance, before making group movements, communicating information about ‘when to go’ among members can allow individuals to coordinate departure times [6,7]; such coordination can reduce the risk of predation and enhance energetic efficiency by allowing individuals to avoid false starts. During activities such as foraging, communicating spatial information about their current positions can minimize the chance of individuals becoming separated from the group [8,9]. The same information can also help regulate spacing of potential foraging competitors, thereby reducing conflicts [8,10]. These examples highlight the significant role that communication plays in coordinating behaviours within groups.

Two central and related questions in collective behaviour are: (i) when is the use of signals as active communication, as opposed to cues as passive communication, necessary, and (ii) which of these forms of communication is most effective? While it seems obvious that acoustic signals should evolve as a means of communication when making collective decisions, movement-based signals or cues (here, we focus on movement cues) still play an important role [11]. For example, humans often acquire social information by observing the movements or behaviours of others that subsequently impact collective decisions [12–14]. In some primates, the direction of departures is often determined by individuals ‘voting with their feet’ [15,16], with individuals coordinating movements following in the footsteps of others [17,18]. In starling murmurations, individuals coordinate their flying direction and speed by copying the behaviour of nearby individuals, rather than relying on acoustic communication [19,20].

What dictates the use of acoustic signals versus movement cues? A group of mammals resting on a hot day would avoid unnecessary activity if they could communicate their preference to leave using acoustic signals rather than movements. By contrast, in highly dynamic flocking birds, using vocalizations to communicate intentions like ‘I want to turn right’ or ‘I want to turn left’ is likely to be unsuitable, being prone to errors owing to signal interference (if many individuals communicate simultaneously), low efficacy (if the sound of flight adds noise) and difficulties in locating the source individual (as the flock is moving fast). Thus, while in some contexts, animals can use acoustic signals to communicate contextual information [21–24], in other contexts, movement cues may be more efficient for coordinating actions. To date, there is no clear conceptual framework that we can draw upon to make predictions about when group-living animals should evolve to use acoustic signals versus movement cues as a means of reaching a consensus and making collective decisions.

Here, we first review how acoustic signals and movement cues are used for coordinating activities in group-living vertebrates, with a specific focus on terrestrial birds and mammals. In this review, we define social behaviour as the extended spatial proximity among individuals, social interactions as any behaviour by one individual that affects or changes the behaviour of another individual, and collective behaviour as the behaviour and movement of groups of animals that result from, or emerge from, social behaviour (maintaining cohesion) and social interactions (the effect of the behaviour of individuals on others). Our emphasis is on collective behaviours in foraging, anti-predator and movement contexts. We then outline a framework that aims to make predictions on whether animals should use acoustic signals versus movement cues when making collective decisions. In developing this framework, we consider informational masking, discrimination ability (e.g. the ability to discriminate small differences) and encoding limitations, as well as trade-offs between these. We also highlight the importance of quorum and quorum-like thresholds in reaching collective decisions. Finally, we discuss potential future avenues of enquiry, including multimodal communication and collective decision-making by mixed-species animal groups.

## 2. The role of acoustic communication in coordinating behaviours

Acoustic communication is often used during coordination of vertebrate groups. Through modifications in frequency, amplitude and call rate, acoustic signals are flexible, allowing them to be used to convey a wide array of information—from the caller’s identity and internal motivations to specific details about external events or objects. Depending on the situation, acoustic signals can either span long distances, reaching all group members almost simultaneously (global communication), or be limited to short-range interactions with neighbouring members (local communication) [3,25]. Specifically, if active space spans the entire spatial extent of the group, as in the case of travel calls and alarm calls, a signaller can directly communicate with all group members. Conversely, acoustic signals that reach only nearby group members, such as soft contact calls, can mediate local interactions and contribute to group coordination.

In this section, we review the literature on acoustic communication used for coordinating activities in social animals, ranging from internal motivational calls, such as contact calls and travel calls, to external referential calls, such as food-associated calls and alarm calls [26]. We also highlight the common use of acoustic communications by group-living animals in maintaining group cohesion during foraging, initiating changes in group behaviours and coordinating cooperative anti-predator responses.

### (a) Acoustic signals for maintaining and coordinating current behaviour

**Figure 1.**
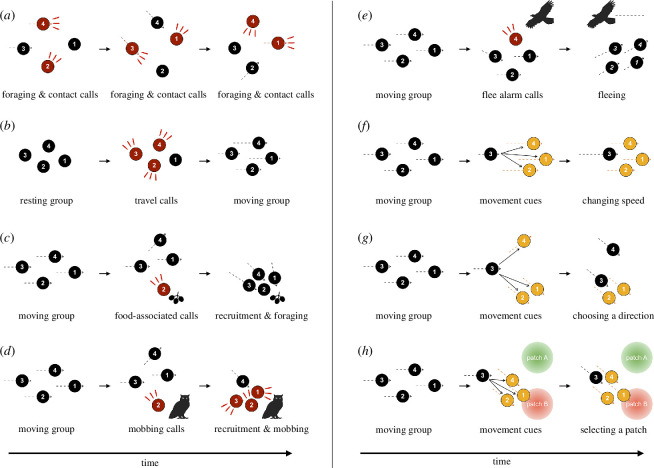
Schematic diagrams illustrating the role of acoustic signals (red circles) and movement cues (yellow circles) in coordinating activities in group-living animals. Behaviours involving the expression of specific actions or the timing of events tend to rely more on acoustic communication for coordination, whereas behaviours related to directional decisions are typically mediated by movement cues. (*a*) Contact calls communicate the location and current state of callers, helping to synchronize individuals’ current behaviours, regulate spacing between them and maintain group cohesion. (*b*) Travel calls communicate the timing of transitions from one group behaviour to another, primarily coordinating departures from a sedentary state. (*c*) Food-associated calls recruit group members to food sources and initiate foraging behaviours. (*d*) Mobbing alarm calls rally group members to collectively mob low-urgency predators, prompting a shift from their normal states to anti-predator actions. (*e*) Flee alarm calls prompt a collective flee response within the group in response to high-urgency predators; once on the move, individuals rely more heavily on movement cues to coordinate their (*f*) speed, (*g*) direction, and (*h*) destination. Black circles indicate individuals, red circles indicate individuals giving acoustic signals and yellow circles indicate movement cues from individuals. Dashed lines indicate movement direction and speed, red lines indicate an individual giving acoustic signals in a specific direction and black thin arrows indicate an individual gaining information from the movement cues of other individuals. Gradient-filled red and green circles indicate different patches.

‘What to do now?’ A crucial decision in synchronizing animal activities is whether to continue current behaviour or switch to a different one. Many socially living animals produce ‘contact’ calls during group foraging, movement and even during resting [8,25,27]. These acoustic signals are believed to facilitate coordinated activities and maintain group cohesion by conveying information about the caller’s location and current motivation to the receivers. For example, southern pied babblers *Turdoides bicolor*, which cooperate to raise young, maintain cohesion and coordinate their foraging by emitting ‘chuck’ calls [28]. These calls are used to spatially organize foragers by maintaining spacing among group members but also keeping the group members together [8], while the rate of call production provides information on the greater need to forage and/or the availability of food [28]. Doing so allows individuals to function effectively as a group, increasing their breeding success [29]. Similarly, meerkats *Suricata suricatta* continuously produce ‘close’ calls during foraging, adjusting the call rates to communicate spatial information between group members, thereby mediating the cohesion of progressively moving groups [10,30]. Moreover, chimpanzees *Pan troglodytes* use ‘rest hoos’ to communicate whether they should resume travelling after a brief stop-over or initiate a prolonged rest period [27]. When the intensity of these vocalizations increases and more individuals respond, they tend to rest for longer durations [27]. Taken together, such calls not only function as ‘location markers’—signalling the caller’s location, regulating spacing between individuals and thereby maintaining group cohesion [8,25]—but they also play a crucial role in conveying an individual’s preferences (i.e. internal motivation) to continue their current behaviour (e.g. foraging or resting) rather than switching to another one (figure 1*a*), thereby facilitating the coordination of group behaviour.

Acoustic communication is also used in another coordinated group activity, sentinel behaviour, which has evolved in some social mammals and birds [31]. Sentinels are individuals stationed in a prominent position that scan for predators and frequently emit specific acoustic signals while the other group members are foraging [32–35]. Sentinels often give soft surveillance calls, providing information about their presence [35]. For example, during sentinel duty, meerkats use sentinel calls to help coordinate their guarding rotation, which substantially increases the foraging time for the other group members [32]. Similarly, in foraging groups of southern pied babblers, sentinels give constant ‘watchman’s calls’ to announce their presence, which allows group members to invest more time in foraging and less time in vigilance [33,35]. Sentinel calls, therefore, not only coordinate vigilance behaviour during foraging but also allow group members to fully focus on foraging without the need for constant vigilance.

### (b) Travel calls in collective departures

‘When to go?’ is one of the most frequent collective decisions that social animals face. Coordinating the timing of departures from a resting site, or moving toward a new foraging patch, is crucial for individuals to maintain the benefits of living in a group [36,37]. Many social animals produce acoustic signals to indicate readiness to travel or to initiate group movements [38]. Recent empirical studies suggest that timing decisions are often mediated by acoustic communication [6,7,39]. These decisions frequently operate under a type of quorum-like process, where a specific acoustic signal has to reach a certain threshold of intensity before the group changes activity [6,7,39]. For instance, among group-living birds, green woodhoopoes *Phoeniculus purpureus* emit calls to initiate group movement and recruit group mates [37], while jackdaws *Coloeus monedula* use vocalizations to coordinate mass departures from communal roosts [7]. Among mammals, African wild dogs *Lycaon pictus* emit specific ‘sneeze’ sounds to switch from resting to moving [6], and meerkats produce ‘moving’ calls to initiate group departure from a foraging patch [39]. In primates, white-faced capuchins *Cebus imitator* make ‘trill’ calls to initiate group movement in stationary troops [40], mountain gorillas *Gorilla berengei berengei* increase their ‘grunt’ rate and more group members call before the transition from resting to moving [41] and similar behaviours are also found in chacma baboons *Papio ursinus* [42]. These studies all highlight the crucial role of acoustic communication in coordinating the timing of transitions from one group behaviour to another (figure 1*b*) and suggest that the increasing intensity of acoustic signals, or having more individuals vocalize simultaneously, can act as a ‘voting’ process [6,39].

### (c) Food-associated calls for attracting group members to food patches

Many social bird and mammal species produce distinctive ‘food-associated’ calls when they encounter food, thereby advertising their location to other group members [43]. In some species, such acoustic signals can even convey specific information about the type, quality or quantity of food, and are hence considered functionally referential [44–46]. Although most research on food-associated calls in animals has focused on their referential functions [44,45] and audience effects [47,48], these acoustic signals can also play a pivotal role in synchronizing and coordinating group foraging [43,49]. Food-associated calls in social bird species can be used to trigger foraging behaviours in others, and such vocalizations are more likely to be produced when individuals cannot exploit the entire resource themselves [50,51]. For example, southern pied babblers produce ‘purr’ calls when they find a rich foraging patch. These calls attract conspecifics, particularly fledglings, to food sources [52]. Willow tits *Poecile montanus* frequently produce long-distance calls when they discover a food source. They use these calls to attract flockmates to foraging patches, especially when separated from conspecifics, suggesting this vocalization helps individuals in coordinating foraging activities, thereby maintaining cohesion [53]. Studies on mixed-species bird flocks support the primary function of these calls being to attract conspecifics when beneficial [54], rather than conveying specific details about the food itself [55]. In primates, spider monkeys (*Ateles geoffroyi*) emit ‘whinny’ vocalizations to attract conspecifics to feeding trees [56], and chimpanzees produce ‘rough grunts’ when they discover food [57]. Unlike in birds, the food-associated calls of chimpanzees can convey information about not only the presence of food but also the size of the food patch and possibly the type of food [58–60], thereby influencing the foraging decisions of the receivers [57]. Finally, bottlenose dolphins *Tursiops truncatus* produce food-associated acoustic signals during social foraging, presumably to coordinate with certain individuals in the group [49]. Taken together, food-associated calls not only recruit conspecifics to food patches but also trigger and synchronize foraging (figure 1*c*).

### (d) Mobbing calls for collective mobbing threat

In many social animals, group members come together to repel external threats. This collective action, known as mobbing, involves two or more individuals synchronously approaching or harassing a threat and is commonly initiated by acoustic signals [61]. Synchronizing mobbing actions is crucial for group-living animals because it can enhance their anti-predator benefits [62,63]. Specifically, the more individuals that participate, the better they can repel potential predators and the lower the risk for each individual [63].

Mobbing calls, similar to food-associated calls, primarily function for recruitment but prompt receivers to switch from their normal states (e.g. foraging and movement) to anti-predator behaviours (e.g. approaching and mobbing calling; figure 1*d*). In many species, the acoustic structure of mobbing calls varies depending on the type or level of predation risk, and can thereby elicit appropriate anti-predator responses in conspecifics [62,64,65]. Although mobbing behaviours have been extensively studied, more recent studies indicate that collective mobbing responses seem to be significantly influenced by the number of calling individuals—the greater the number of simultaneous callers, the more likely it is for group members to participate in the mob [66,67]. This implies that collective mobbing might involve quorum-like decision-making. For example, jackdaws assess the number of conspecifics involved in initiating mobbing events by recognizing individually distinctive recruitment calls [67,68]. Playback simulations found that three or five callers attracted more individuals than a single caller, showing that jackdaws can recognize the number of callers from these vocalizations and use that information in deciding their participation in mobbing events [67]. Similarly, in great tits *Parus major*, the decision on whether to respond to conspecific mobbing calls—like approaching threat signals or emitting their own mobbing calls—is influenced by the number of callers [66]. Specifically, great tits respond more strongly to the mobbing calls of three callers than to one caller, although the mobbing calls of five callers did not elicit an even stronger response. Additionally, spotted hyenas *Crocuta crocuta* use long-range recruitment vocalizations, known as ‘whoops’, to coordinate their collective defence of resources, territories and against threats [69,70]. Although no direct playback experiments indicate that a greater number of callers intensifies mobbing responses, the number of hyenas and presence of social allies and kin are shown as important factors in their decision to mob predators. In conclusion, mobbing calls play an important role in coordinating collective anti-predator behaviours in social animals, but sensory limitations (following Weber’s Law; see §4a(ii)) might constrain the ability for individuals to perceive the complete gradient of information available as the number of callers increases [66,67].

### (e) Alarm signals for avoiding predators

Flee alarm calls, commonly used to coordinate anti-predator behaviours, often prompt receivers to shift from their current activities to vigilance or flee (figure 1*e*). In social animals, collective vigilance and coordinated anti-predator responses are key benefits of group living [71–74]. These benefits are amplified when individuals can efficiently communicate and transfer information about danger. Alarm calling—the production of specific acoustic signals upon detecting a predator—is particularly effective because it can quickly alert all nearby group members, even if other individuals are not currently vigilant or are out of sight [25,75]. For example, common starlings *Sturnus vulgaris* are more likely to emit alarm calls in long-grass habitats than in short-grass habitats, suggesting that their alarm calls are crucial for coordinating group anti-predator responses when visual cues are impractical [76]. Additionally, in some species lacking vocal alarm calls, specific sounds can be acoustic alarm signals. For instance, crested pigeons *Ocyphaps lophotes* produce distinct ‘whistle’ alarm signals using their modified wing feathers, triggering rapid fleeing behaviours in foraging groups [77,78].

Referential alarm call systems, which represent a more complex form of alarm communication, enable receivers to respond more effectively and appropriately, even in the absence of direct cues from the threat itself [79]. These types of alarm calls can convey predator-specific information, such as predator type [80,81], size [61,65], behaviour [82] and urgency level [83,84], thereby prompting fine-scale coordinated anti-predator responses. Such systems have been documented in a variety of group-living mammal and bird species [79,85,86]. A classic example is vervet monkeys *Chlorocebus pygerythrus*, which give different types of alarm calls to snakes, leopards and eagles, and other group members respond appropriately to playback of those calls, such as running into trees after leopard alarms and looking up and running into cover after eagle alarms [80]. Such specific information can prompt fine-scale coordinated anti-predator responses among group members, enhancing their chances of survival, as escaping in the wrong direction or responding inappropriately can potentially lead to fatal mistakes [87,88]. Similar behaviours can be found in many social primates, such as Diana monkeys *Cercopithecus diana* [89], Campbell’s monkeys *Cercopithecus campbelli* [90,91] and non-primate mammals, e.g. meerkats [23] as well as birds, e.g. chickens *Gallus gallus* [44]. Furthermore, referential alarm calls in some species can simultaneously convey more than one type of predator-related information. For instance, meerkats combine information by producing alarm calls depending on predator type as well as varying acoustic structure to convey urgency information [23,92], and Siberian jays *Perisoreus infaustus* produce alarm calls that encode predator behaviour and not just taxonomic categories [93]. These examples illustrate that social animals commonly evolve complex alarm call systems and possess the capacity to produce a wide variety of acoustic signals that convey referential information, ultimately coordinating fine-scale anti-predator behaviours within groups.

## 3. The role of movement cues in coordinating behaviours

Once on the move, individuals within a group have to constantly coordinate their directions, speed and next destination. Understanding how these individuals coordinate their movements can be challenging, particularly when there are no clear starting and stopping points during their traveling. Although mammals and birds commonly use acoustic communication to coordinate activities, empirical research suggests that directional decisions, such as those made by groups on the move or when choosing a destination, are predominantly mediated by movement cues [15,42,94,95]. For example, olive baboons *Papio anubis* use a simple rule, ‘voting with their feet’ by making directed movement initiations, to decide on movement direction. Specifically, individuals make a short, directed movement towards their preferred movement direction, and group members tend to follow the direction with the most initiators (i.e. votes) [15]. These types of movements are likely to be widespread and appear to provide an important cue for others to follow. For example, while mountain gorillas increase ‘grunt’ vocalizations to reflect a readiness to move [41], dominant silverbacks always take the lead in a certain direction, after which other group members follow [96]. Similarly, meerkats use ‘moving’ calls to increase movement speed; however, these calls have not been associated with changes in direction, suggesting that influencing movement direction may require an additional cue (likely visual) to specify the intended direction [39,94].

While animals can communicate their intentions through directed movements (often a straight movement at intermediate speed) [15], there is also a growing body of evidence that decisions can emerge through simple, local interactions among neighbours. For example, in flocks of starlings, individuals pay attention to around eight local neighbours, coordinating their speed and turn to maintain consistent spacing [19]. Simulations propose that such topological (or zonal—where individuals avoid, align and are attracted to conspecifics at different distances) interaction rules can allow groups to make effective collective decisions, such as choosing between two foraging patches, even when only a fraction of group members are knowledgeable [97]. Thus, there is significant scope for collective decisions—especially those such as simple navigational tasks—to be reached without any active communication, but instead based on simple rule-based responses to cues.

Our understanding is limited about how and when animals make consensus decisions regarding a specific destination, and it remains unclear whether (or when) all group members become aware of the final destination. For example, how often do particular paths lead to specific resources, and do animals learn these associations? If animal groups repeatedly re-use the same locations—for example, for foraging, drinking or resting—then it is likely that directional movements are interpreted not only in terms of their direction but also the ultimate goal of the movement. In certain instances, the movement direction and destination can be also determined by specific group members. For example, older African elephants (*Loxodonta africana*) play a key role in coordinating group movements [98,99], and killer whales (*Orcinus orca*) heavily rely on older females to lead collective movements in hunting grounds [100] when conditions are poor and resources are scarce. Thus, there is much to be discovered in terms of how much information is encoded about movement objectives and in who is engaging in these actions.

More broadly, the importance of non-acoustic cues in coordinating social behaviours remains much less studied than acoustic signals, likely owing to the more challenging task of quantifying and recording visual cues. Yet, visual signals are widely used in a range of other social behaviours. For example, many mammals and birds use facial signals, such as teeth-baring [101] or beak gaping [102], as a low-cost display of subordinance or dominance. In chimpanzees, one such display—lip smacking—has been shown to increase the length of grooming bouts and the probability that grooming would be reciprocated [103]. In canids, play bows have been shown to promote playful interactions, which could otherwise be misinterpreted as aggressive interactions [104]. While in most of these examples, the signals are used in dyadic interactions, the importance of movements as a trigger for responses by others is likely to have been underappreciated. For example, walking out from the core of a social group represents an unusual behaviour that catches the attention of others. Thus, movements can be very strong signals, and these can represent clear intentions.

## 4. Predicting the relative roles of acoustic signals and movement cues in collective behaviours

Within a group, individuals rely on different forms of social information to coordinate their behaviours across a range of contexts. Understanding the modalities used to produce and acquire signals or cues that coordinate collective actions is crucial for identifying the mechanisms underlying the evolution of social groupings. Here, we outline a framework to determine the types of collective behaviours that are more likely to use acoustic communication or movement cues for coordinating actions and to assess their significance and limitations.

Our review identifies that acoustic signals are likely to be more prevalent for some decisions, and in some environments, than visual movement cues. For instance, behaviours involving the timing of events or expression of specific actions, such as deciding when to depart (figure 1*b*) or whether to mob a predator (figure 1*d*), likely rely more on acoustic signals. Furthermore, acoustic signals are valuable in situations where intended receivers are engaged in other activities [105,106], like foraging or resting, or to convey urgent information about the caller’s intentions or nearby threats. Similarly, sounds are more effective in environments where visual signals might be difficult to perceive because of the habitat or poor lighting conditions. In contrast to acoustic signals, movement cues are likely to be important for decisions involving complex choices with multiple options, when more individuals are involved in making the decision, and when dynamic spatial and direction information is critical [15,16]. Our framework, below, captures how these patterns reflect the limitations in producing and acquiring signals and cues, different trade-offs that individuals face during decision-making and ways in which signals and cues can be aggregated.

### (a) Sensory limitations

#### (i) Informational masking

As the number of individuals simultaneously contributing to a given collective decision increases, it becomes increasingly challenging to recognize and extract information from the signals or cues. Acoustic signals are vulnerable to interference from environmental noises [107,108], which in social groups include sounds from conspecifics [109,110]. In noisy social environments, for example, humans frequently face the ‘cocktail party problem’, which refers to the difficulty that humans encounter when recognizing speech in such settings, when acoustic signals often overlap in frequency and timing, resulting in direct acoustic interference and informational masking [109,111]. Group-living animals communicating acoustically in social aggregations also encounter cocktail-party-like challenges, particularly when group members produce different types of acoustic signals simultaneously [112–115]. For example, when bottlenose dolphins are in groups larger than 15 individuals, their whistle rates decrease [114]. Similarly, when many bats emit echolocation calls simultaneously, detecting and recognizing the echoes generated by one’s own calls becomes more challenging [112,116]. In meerkats, adults reduce their close-call production when pups are foraging with the group. This reduction is likely owing to the loud begging calls from pups, which can mask the adults’ softer close calls [113]. These examples show that while animals can adapt their acoustic behaviours to solve cocktail-party-like problems, the number of individuals emitting acoustic signals simultaneously will influence the efficiency of acoustic communication. Thus, there is likely to be a potential upper limit to the number of acoustic signals that can transmit information effectively at any one time, because when acoustic signalling increases, the potential for interference from other signals also rises [114].

Movement cues appear not to suffer as severely as acoustic signals from information masking. They can be used to coordinate movement in very large groups, such as in murmurations of starlings, because the interactions are limited to a local set of neighbouring individuals, with the collective behaviour scaling up from these dyadic interactions to affect the behaviour of the entire group. While local visual perception can limit the ability of single individuals to broadcast a signal to entire groups (in large, dense or widely distributed groups), studies of schooling fish have found that relatively few individuals are needed to lead very large groups [117]. Thus, as groups become larger and more individuals are involved in making any given decision, we predict that movement cues will become more important than acoustic signals (with some exceptions; see §4c).

#### (ii) Assessment of number and intensity

**Figure 2.**
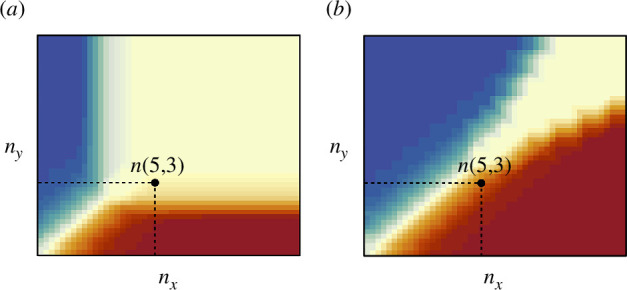
Schematic diagrams depicting the discrimination ability for (*a*) acoustic signals and (*b*) visual cues, as a function of the difference in the number of individuals communicating each of two preferences (i.e. Weber’s Law). Dark red and dark blue represent high probabilities of selecting either option X or option Y, respectively, corresponding to which option has the highest number of ‘votes’. The yellow area represents where individuals cannot reliably discriminate between the relative number of ‘votes’ and consequently choose at random. In the illustrated example, the greater discrimination ability allows the group to select option X when there are five versus three visual cues for X versus Y, but to choose at random when there are five versus three acoustic signals because they cannot discriminate which option has more votes. The design of this figure is based on Arganda *et al*. [119].

As the absolute number of signals or cues increases, individuals also face the challenge of distinguishing the relative differences in stimuli, as revealed by Weber’s Law. Weber’s Law suggests that animals usually compare stimuli based on proportional differences in stimulus magnitude rather than absolute differences [118,119]. That is, as the quantity of different stimuli increases, the comparison of absolute differences between these becomes more difficult. This means that the difference needs to be greater in order for individuals to identify which is the larger amount when there are more stimuli (figure 2). Thus, even without information masking, animals can show limitations in distinguishing the difference in the number or intensity of stimuli as the number of contributors to a decision increases.

Acoustic signals appear to be more susceptible to ‘Weber’s Law’ effects than visual cues and signals. Many bird and mammal species have been shown to possess considerable numerical abilities, at least when assessing visual cues [120–125]. For instance, jungle crows *Corvus macrorhynchos* consistently choose the larger quantity whether in familiar smaller sets (e.g. 3 versus 5) or in novel larger comparisons (e.g. 5 versus 7) [122]. Semi-free-ranging rhesus monkeys *Macaca mulatta* can naturally discriminate and choose containers with more apple slices in comparisons up to three versus five slices but struggle with higher quantities [123]. However, acoustic stimuli appear more vulnerable to Weber’s Law than visual stimuli. For instance, in jackdaws, a single mobbing caller recruited fewer individuals than more callers, but there was no significant difference in the numbers recruited to three compared to five callers [67]. Similar patterns occur in playback experiments of great tits’ mobbing calls, meerkats’ moving calls and female lions’ (*Panthera leo*) roaring vocalizations [39,66,126]. These findings imply cognitive limitations in distinguishing the number of acoustic signals above a certain threshold [67], although there may also be a role for informational masking (above) or the cost borne by group members if they do not accurately select the majority. The difficulty in discriminating small differences as quantities become larger (or the number of options becomes greater) suggests a role for Weber’s Law in predicting the relative importance of acoustic versus visual signals and cues in coordinating behaviours.

In addition to the increased visual discrimination ability of animals (relative to acoustic discrimination), movement-based decisions can also act to reduce the total number of individuals that one group member can perceive (e.g. its local neighbours). Doing so reduces the effect of Weber’s Law that is faced by individuals involved in a decision using movement cues, with the individual-level decisions being aggregated up through the collective to identify the majority decision even in very large groups [97].

#### (iii) Encoding limitations

Acoustic signals often convey information about an animal’s state or motivation, or external objects and events, but movement cues appear better at conveying specific information about direction and speed. Acoustic signals can often communicate about the state of the caller, such as fear or hunger, and motivation, such as intent to fight or defend a territory [25]. Referential calls can also communicate about external objects or events. For example, Japanese tits (*Parus minor* ) emit alert calls to warn conspecifics about predators, while they produce recruitment calls to attract conspecifics in non-dangerous situations [127,128]. These two types of calls are combined into alert-recruitment call sequences when mobbing predators, a capability similarly also observed in southern pied babblers [24]. Referential alarm calls can, for example, indirectly convey directional and distance information. Vervet monkeys look ‘up’ when they hear eagle alarms and look ‘down’ for snake alarms [80], showing that these calls provide information about the direction of threat. White-browed scrubwrens *Sericornis frontalis* vary their aerial alarm calls depending on the distance to a predator in flight, which conveys information about the proximity of danger to conspecifics [84]. However, even though acoustic referential signals convey limited directional and distance information, there is no evidence that acoustic signals can communicate specific directional information, such as ‘left’ or ‘right’. Movement cues, by contrast, can provide detailed information about specific directions. Aside from dynamic movements in mobbing groups (above), an individual initiating movement along a particular animal track provides unambiguous information about its directional preference. The speed and directedness of the movement may also encode information about the strength of this preference [15]. However, whether particular movement cues can convey broader contextual information, such as a preference to follow a given path to reach water versus a food patch, remains largely unknown.

### (b) Key trade-offs underpinning the use of different modalities in collective behaviours

Animal collective behaviour, specifically decision-making, is largely governed by two key trade-offs: salience (conspicuousness) versus complexity and speed versus accuracy [129,130]. Understanding how sensory limitations contribute to these trade-offs can also provide insight into the relative importance of acoustic signals versus movement cues in animal collectives.

#### (i) The salience–complexity trade-off

Effective communication requires precision in the information being conveyed, but precision increases the complexity of a signal or cue. For example, distinguishing preferences between different types of food requires more different signals relative to simply communicating a preference for feeding [43]. As complexity increases, signals or cues need to: (a) be more distributed across the communication space (e.g. across the frequency spectrum) and (b) become more different from one-another. This not only requires greater cognitive ability (potentially increasing decoding errors), but it also means that the salience of these signals or cues is necessarily decreased. Consider the difference between shouting ‘stop’ versus ‘please finish eating’. The latter contains more information but is less salient, and would be more difficult to discern in a busier acoustic environment owing to the increased potential for information masking. By contrast, ‘stop’ is easy to receive and interpret but conveys no specific information. Thus, the salience of acoustic signals will decrease if they are used to convey more information, and at some point, will become lower than movement cues or signals.

#### (ii) The speed–accuracy trade-off

Faster decisions are often made using information acquired only from one or a few individuals, meaning that they are more prone to errors because they do not benefit from information pooling. By contrast, decisions involving information from more individuals can take much longer to resolve [131]. This introduces a speed–accuracy trade-off in collective decision-making. In general, in more urgent situations—such as an imminent attack by a predator—decisions can made using information from just a few individuals, and more effectively made by global (i.e. acoustic) signals. By contrast, if the importance is that the correct decision be made (e.g. selecting a migration route), then preferences should be pooled over a larger number of individuals. The latter should favour visual modes of communication, as this maximizes individuals’ abilities to discriminate smaller differences in which option has the majority of individual preferences.

### (c) Quorum thresholds as a general principle for aggregating preferences in collective behaviours

Quorum decisions involve making a choice based on reaching a threshold number (or intensity) of individuals that are engaged in an activity or signalling a preference [132]. Thus, quorums are most often considered in situations where the decision involves a change in behaviour as opposed to choosing between a large number of options (e.g. directional movement decisions based on a majority rule). For example, groups of vulturine guineafowl *Acryllium vulturinum* leave food patches when—on average and independent of group size—13 group members have initiated movements away from the patch [95], presumably because waiting for an absolute majority becomes too costly for group members. However, these concepts are not diametrically opposed, as majority-based decisions can also be made when a given ‘sub-majority’ is reached. For example, baboons will follow when there are fewer initiators if these all agree in their direction but require more initiators if there is greater disagreement among initiators [15]. Here, we briefly highlight the importance of quorum- and quorum-like thresholds in reaching collective decisions.

**Figure 3.**
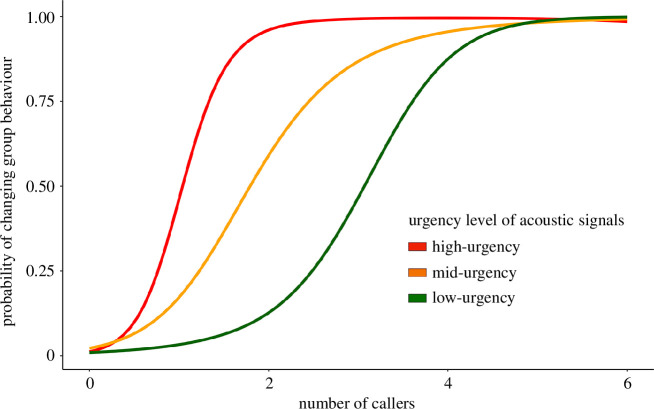
Schematic diagrams illustrating the probability thresholds for changing group behaviour based on varying levels of urgency in acoustic signals. The red line represents high-urgency acoustic signals, such as flee alarm calls; the orange line represents mid-urgency signals, such as mobbing calls; and the green line represents low-urgency signals, such as travel calls.

Quorum thresholds can differ based on the context, influenced by the level of urgency conveyed by the information. For example, in high-urgency situations like imminent threats, a group might have a lower quorum threshold to coordinate actions swiftly (figure 3). High-urgency signals, like aerial alarm calls, from just one individual can be sufficient to initiate collective escape actions (figure 1*e*). By contrast, for less-urgent situations like mobbing, the collective response may necessitate signals from a greater number of individuals (figure 1*d*), resulting in a higher quorum threshold for collective action. Thus, where the threshold is set will have a major impact on the speed at which decisions are made.

Quorum thresholds can also be influenced by the reliability of information. As the group size grows, for example, false alarms can become more frequent [133], thereby reducing the accuracy of decisions. This suggests an interaction between the speed–accuracy and the salience–complexity trade-offs in determining where the threshold is set. For example, using movement (e.g. a take-off flight) as a cue for an attack represents a relatively simple cue (high salience), but individuals make movements for a range of reasons (e.g. in response to social interactions), making it more inaccurate (requiring a higher threshold to avoid false positives). A more complex alarm call that is specific to a predation threat is also very salient, but less prone to false positives (requiring a lower threshold to avoid false positives). However, if alarm calls become too complex and begin overlapping with other (e.g. social) signals, they could also be prone to suffering from false positives or be too difficult to decode (thereby reducing accuracy). Thus, the interactions between these trade-offs warrant more detailed experimental investigations.

## 5. Collective decision-making in vertebrate vs non-vertebrate organisms

While our focus in this paper has been on vertebrate decision-making, collective action can be expressed by most other organisms, including invertebrates and bacteria [134–136]. For example, plagues of locusts can move as a cohesive group over continental scales [134], and both invertebrates and bacteria are capable of quorum sensing [135,137]. These organisms can benefit from the emergent properties of collectives to most effectively exploit their environment (e.g. slime moulds can solve two-armed bandit problems [138]). While there are many distinctions between collectives of vertebrates versus those of non-vertebrates (e.g. the stratified relationships within social groups), many of the same biases are likely to be a feature of collective behaviours of both. For example, both vertebrates and non-vertebrates decrease in their ability to discriminate between numerical differences as the number of individuals involved in a collective behaviour increases (Weber’s Law). One notable factor in most invertebrate and bacterial systems is that they rarely use acoustic cues or signals, and instead use very local modes of communication—such as cell-to-cell signalling in bacteria [139], pheromones in ants [140] or physical cues in locusts [141,142]. The reasons for this—sensory limitations—are likely similar to why similar local cues are used in large groups of vertebrates, like starlings [19] and fish [143]. While our review is not focussed on collective decision-making in non-vertebrates, further consideration of the similarities and contrasts between vertebrate and non-vertebrate social organisms should shed more light on how ecology, cognitive and sensory limitations have shaped the evolution of collective actions.

## 6. Outstanding issues and future directions

In this review, we present a framework to identify which types of collective behaviours likely rely on acoustic signals or movement cues for coordination, while also assessing their significance and limitations. Nonetheless, the predictions mentioned necessitate further exploration, such as exploring: (i) how sensory limitations, shaped by the effects of ‘Weber’s Law’, influence coordination behaviours and (ii) how different types and reliabilities of information influence the quorum-like thresholds needed to reach a group consensus. These concepts can further be extended to more complex situations, such as multimodal signalling and collective behaviours within mixed-species animal groups.

### (a) Multimodal communication

Each sensory modality has its own strengths and limitations, but combining multiple senses can enhance signal efficiency and potentially facilitate group consensus decisions. Animals, particularly birds and mammals, commonly rely on auditory and visual senses to coordinate their immediate activities. Generally, hearing has a high temporal resolution, which is beneficial for judging timing and estimating distance. On the other hand, vision has a greater angular resolution, making it more effective for determining the number of objects, direction and dynamic cues in groups. These attributes can supplement each other, and thus, enhance signal and communication efficiency between senders and receivers [144,145]. For example, when attempting to initiate a collective movement, white-faced capuchins display various behaviours, such as emitting ‘trill’ vocalizations, giving back-glances and/or moving at a slow speed, to increase the probability of a successful departure [40,146]. Also, chickens produce food-associated calls that are typically accompanied by a visual display, creating a multimodal signal, with each modality as a backup signal to potentially enhance signal efficiency [147]. Alarm calls can quickly convey ‘alert’ information to receivers, while by observing the caller’s subsequent behaviours (e.g. direction of scanning or escaping), receivers can refine the information, such as the specific direction of an approaching predator, and ultimately respond more appropriately and accurately. Thus, while acoustic signals likely function as an ‘initiation', complementary information from other senses can enhance the signal’s efficiency and clarity, leading to more efficient coordinated collective actions. The integration of signals from multiple sensory modalities, like acoustic and visual, remains largely unexplored in the context of collective behaviour. Further studies investigating how modalities interact within the context of, for example, making collective decisions should be encouraged [5].

### (b) Collective behaviours in mixed-species animal groups

Do animals in mixed-species groups use the same mechanisms to coordinate collective behaviours as they do in single-species groups? While our current understanding is still limited, the mechanisms seem to be similar [148]. Mixed-species animal groups, comprised of multiple species that forage and move together in a coordinated manner, are commonly observed across diverse taxa and habitats [149]. Individuals from different species coordinate their activities to maximize group benefits, such as reducing predation risk and enhancing foraging efficiency. Previous studies indicate that interspecific acoustic communication can play an important role in coordinating mixed-species group behaviours, particularly in birds [150,151]. Similar to single-species groups, contact calls maintain mixed-species group cohesion [152–154], food calls attract both conspecific and heterospecific members to food patches [53], mobbing calls coordinate collective mobbing behaviours across species [155–157] and aerial alarm calls elicit heterospecific escape responses [158,159]. However, our understanding of how mixed-species groups determine departure times, movement directions, speed and destinations is still limited. Such coordination might largely be influenced by specific species, as seen with many ‘leader’ species in mixed-species bird flocks [149,160]. Future studies exploring how acoustic signals and movement cues are used for group movement coordination, and investigating how different species reach a consensus decision (e.g. through a quorum or non-shared process) can help unravel the evolutionary mechanisms driving the formation of complex mixed-species animal groups.

## Data Availability

This article has no additional data.
